# A descriptive analysis of medical health services utilization of Veterans living in Ontario: a retrospective cohort study using administrative healthcare data

**DOI:** 10.1186/s12913-016-1596-y

**Published:** 2016-08-04

**Authors:** Alice B. Aiken, Alyson L. Mahar, Paul Kurdyak, Marlo Whitehead, Patti A. Groome

**Affiliations:** 1 School of Rehabilitation Therapy, Queen’s University, Kingston, ON Canada; 2 Department of Public Health, Queen’s University, Kingston, ON Canada; 3 Centre for Addiction and Mental Health, Toronto, ON Canada; 4 Institute for Clinical Evaluative Sciences, Toronto, ON Canada

**Keywords:** Veterans, Health services, Military personnel, Administrative healthcare data

## Abstract

**Background:**

Health services utilization by Veterans following release may be different than the general population as the result of occupational conditions, requirements and injuries. This study provides the first longitudinal overview of Canadian Veteran healthcare utilization in the Ontario public health system.

**Methods:**

This is a retrospective cohort study designed to use Ontario’s provincial healthcare data to study the demographics and healthcare utilization of Canadian Armed Forces (CAF) & RCMP Veterans living in Ontario. Veterans were eligible for the study if they released between January 1, 1990 and March 31, 2013. Databases at the Institute for Clinical Evaluative Sciences were linked by a unique identifier to study non-mental health related hospitalizations, emergency department visits, and physician visits. Overall and age-stratified descriptive statistics were calculated in five-year intervals following the date of release.

**Results:**

The cohort is comprised of 23, 818 CAF or RCMP Veterans. Following entry into the provincial healthcare system, 82.6 % (95 % CI 82.1–83.1) of Veterans saw their family physician at least once over the first five years following release, 60.7 % (95 % CI 60.0–61.3) saw a non-mental health specialist, 40.8 % (95 % CI 40.2–41.5) went to the emergency department in that same time period and 9.9 % (9.5–10.3) were hospitalized for non-mental health related complaints. Patterns of non-mental health services utilization appeared to be time and service dependant. Stratifying health services utilization by age of the Veteran at entry into the provincial healthcare system revealed significant differences in service use and intensity.

**Conclusion:**

This study provides the first description of health services utilization by Veterans, following release from the CAF or RCMP. This work will inform the planning and delivery of support to Veterans in Ontario.

**Electronic supplementary material:**

The online version of this article (doi:10.1186/s12913-016-1596-y) contains supplementary material, which is available to authorized users.

## Background

Health services utilization by Veterans may be different than the general population. Occupational conditions, requirements and injuries likely determine differences in healthcare needs among Veterans. In Canada, members of the Canadian Armed Forces (CAF) transition from a federally-run and highly specialized healthcare system, the Canadian Forces Health Services (CFHS) to a provincial, publicly-funded healthcare system at the end of their career. Until April 2013, members of the Royal Canadian Mounted Police (RCMP) used the provincial healthcare system in Canada while serving; however they were considered non-provincial residents and their health services use was billed to the federal system [1–3]. Veterans of the CAF and the RCMP may be supported by additional health benefits from Veterans Affairs Canada (VAC); however, only 35 % of Regular Forces Veterans report receiving benefits [4]. Approximately one third of VAC clients are war service Veterans, one third are surviving families of Veterans, 27 % are CAF members or Veterans, and 3 % are RCMP [5]. The transition to civilian life is already a difficult adjustment for approximately 25 % of Veterans [6] and this difficulty may be exacerbated by challenges inherent in navigating appropriate health services within the provincial system after transition from the federal system.

The unique exposures of an individual’s career in the CAF or RCMP likely influence subsequent health care needs and patterns of health services use as a civilian. A study of mortality occurring within the Canadian military concluded that almost 50 % of deaths were caused by medical disease, with 35 % resulting from preventable or modifiable behaviours such as smoking and alcohol use [7]. Studies from VAC report that 17 % of Veterans who served full time in the military reported being a daily smoker, and 25 % met criteria for being a heavy drinker [4]. The Life After Service Studies from VAC concluded that Canadian Veterans have a higher prevalence of many self-reported chronic health conditions than the general population [4]. Over 70 % of Regular Forces Veterans reported having one or more chronic physical condition and 24 % reported a chronic mental health condition [4]. However, patterns of health services utilization related to these high rates of medical problems in Canadian Veterans have not been previously described or been available for study. This information is needed to understand the transition to civilian life that includes accessing healthcare in a civilian health care system, and to plan future health services use.

Longitudinal health services utilization data following Canadian Veterans from the time that they are released has not been reported until now. Data collected by VAC through the Life After Service surveys have been the only source of information on health services utilization of Veterans in Canada [4, 6]. This information is limited because it is cross-sectional and only provides details about private health insurance (prescription, dental, vision) and access to a regular family doctor (self-reported rather than actual claims data) [4].

Our group has recently reported on the development of a resource of longitudinal data to study Veterans residing in Ontario [8]. This resource utilized Ontario’s administrative healthcare databases and has incredible research potential to augment existing survey data collected by Veterans Affairs Canada (VAC) [4, 6]. The purpose of this study is to describe the non-mental health services utilization of Veterans in Ontario over time, stratified by age at release.

## Methods

### Study design & population

This was a retrospective cohort study designed to use Ontario’s provincial healthcare data to study the healthcare utilization of Veterans entering the provincial healthcare system in Ontario. A description of this cohort has been published [Mahar et al., *Journal of Military, Veteran and Family Health*, *in press*]. This study is a descriptive analysis of the non-mental health services utilization of the Canadian Veteran population in Ontario. Ethics approval for this study was obtained from the Queen’s University Health Sciences and Affiliated Teaching Hospitals Research Ethics Board. Individual patient consent was not required. The Institute for Clinical Evaluative Sciences is a s. 45 Prescribed Entity under Ontario’s privacy law (PHIPA) enabling us to study the health and health outcomes of individuals for the purpose of analysis or compiling statistical information with respect to the management of, evaluation or monitoring of, the allocation of resources to or planning for all or part of the health system.

Individuals were eligible for the study if they had registered for Ontario Health Insurance Plan (OHIP) coverage between January 1, 1990 and March 31, 2013 and provided evidence of a previous career in the CAF or RCMP. The date of OHIP registration was used as the index date for entry into the cohort and approximated the service release date. Individuals were followed from the time they left the CAF or RCMP until death, the end of OHIP eligibility (e.g., moved out of province), or the end of the follow-up period (March 31, 2013).

### Identifying veteran status

In Canada, the healthcare of Canadian Armed Forces (CAF) personnel and RCMP officers is federally regulated. CAF personnel are provided care within a specialized military system separate from the provincial healthcare systems. Until April 1, 2013, RCMP officers were considered non-provincial residents and their health services use was billed to the federal government [1–3]. In Ontario, the Ministry of Health and Long Term Care (MOHLTC) tracks registration for provincial healthcare coverage for previous CAF and RCMP members using specific administrative codes on the OHIP application form and this is stored alongside the health card number. This form provides the necessary documentation on service history to waive the standard three-month waiting period for provincial healthcare coverage. For this study, Veterans were defined as CAF and RCMP service leavers who provided evidence to the MOHLTC about their career history. The MOHLTC provided the authors with the anonymized list of people with an administrative CAF and RCMP service code linked to their health card number, as well as career start and end dates. This information was linked to the Institute for Clinical Evaluative Sciences (ICES) data holdings using unique encoded identifiers and analyzed at ICES. Details on cohort creation and demographics are provided elsewhere [8, 9].

### Data sources & variables

The following administrative healthcare databases housed at ICES were linked to the MOHLTC Veteran data to describe the provincial health services utilization of Ontario Veterans over time. The Canadian Institute for Health nformation Discharge Abstract Database (CIHI-DAD), the National Ambulatory Care Reporting System (NACRS), OHIP and the Registered Persons Database (RPDB).

The CIHI-DAD is a repository of inpatient admission and discharge data (except hospitalizations occurring in designated psychiatric beds as of 2005), including all medical inpatient admissions in the province. It was used to identify non-mental health related hospitalizations and cumulative inpatient stay. The NACRS database is a warehouse of outpatient hospital visit data, including emergency department, oncology and dialysis. It was used to identify non-mental health related emergency department (ED) visits. The OHIP database is a repository of physician billing data for healthcare encounters and procedures. It was used to identify non-mental health related family physician visits, as well as to determine provincial healthcare coverage eligibility dates for the cohort. Standard ICES definitions were applied to the data to measure these healthcare encounters and preventative health behaviors [10]. Physician and emergency department visits, and hospitalizations related to mental disorders will be reported elsewhere. The Registered Persons Database (RPDB) provided demographic information, using data supplied by the MOHLTC and supplemented by other provincial data holdings at ICES.

### Non-mental health services utilization

In this study, we exclusively evaluated medical and non-mental health services utilization among Veterans in Ontario. Non-mental health hospitalizations were defined as admissions that did not have a most responsible diagnosis of a mental disorder (e.g., all ICD-10 F codes). They were measured as a dichotomous variable (hospitalized yes/no) and as a count (number of hospitalizations). Cumulative inpatient stay was defined as the total number of days spent in hospital, during the study timeframe, regardless of the number of re-admissions or location of stay. ED visits were measured as a dichotomous variable (ED visit yes/no) and as a count (number of ED visits). To measure non-mental health related ED visits, visits with a most responsible diagnosis of a mental disorder (all ICD-10 F codes) were excluded. Family physician and specialist physician visits were studied separately. Family physician visits were restricted to defined as visits to doctors with specialties in family medicine, family medicine/emergency medicine. Specialist visits were defined as visits with any other specialist, excluding psychiatry. Both were measured as dichotomous variable (yes/no) and count (number of visits). Mental health related family physician visits were excluded according to a previously validated algorithm [10, 11]. Healthcare encounters with physicians for mental disorders were also excluded from this description using ICD-9 codes.

### Data analysis

A description of all resource utilization measures is presented using frequencies and 95 % confidence intervals for categorical variables. One-sample proportion confidence intervals for categorical variables were calculated using the normal approximation to the binomial distribution. Mean, standard deviation (SD), median and interquartile range (IQR) are provided for continuous and count data.

All descriptive statistics were stratified by five year time intervals following release (0–5 years, 5–10 years, 10–15 years, and 15–20 years) to investigate temporal patterns in medical healthcare utilization within the cohort. Baseline was considered the first five years following release. This is similar to how the results of a panel study are presented, integrating both cross-sectional and cohort designs to present snapshots of a cohort over time [12]. To determine if differences existed across time, 95 % confidence intervals were used, corresponding with a *p* <0.05.

We a priori anticipated that a cohort effect by age at release would exist. To explore possible patterns in medical healthcare utilization, stratified descriptive statistics were also presented by age at release categories. To determine if differences existed across age categories, 95 % confidence intervals were used, corresponding with a *p* <0.05. Cell sizes less than 6 are suppressed according to ICES privacy regulations. All descriptive analyses were performed using SAS 9.3 (Cary, North Carolina, USA).

## Results

**Fig. 1:**
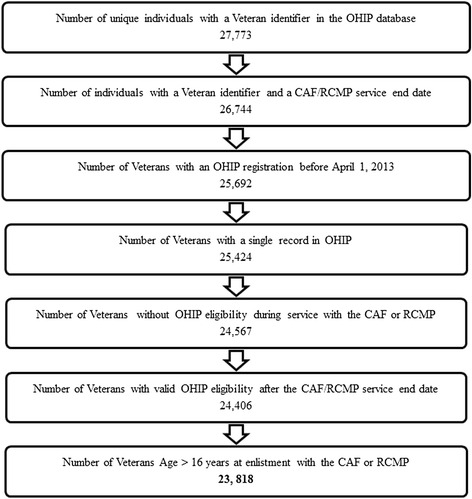
Description of cohort eligibility and exclusion criteria

The cohort is comprised of 23, 818 CAF or RCMP Veterans who lived in Ontario and provided documentation of their service history when applying for an OHIP card (n excluded = 3,955) (Fig. 1). The average follow-up time was 9.33 years (SD 6.13). The average age of the cohort on entry into the Ontario healthcare system was 53.6 years (SD 2.53) and approximately 15 % of Veterans were female. The majority of Veterans resided in areas with the top three highest median community income quintiles and in an urban area, specifically in the the South East and Champlain Local Health Integration Networks (LHINs), two of the health service regions of Ontario.

**Fig. 2:**
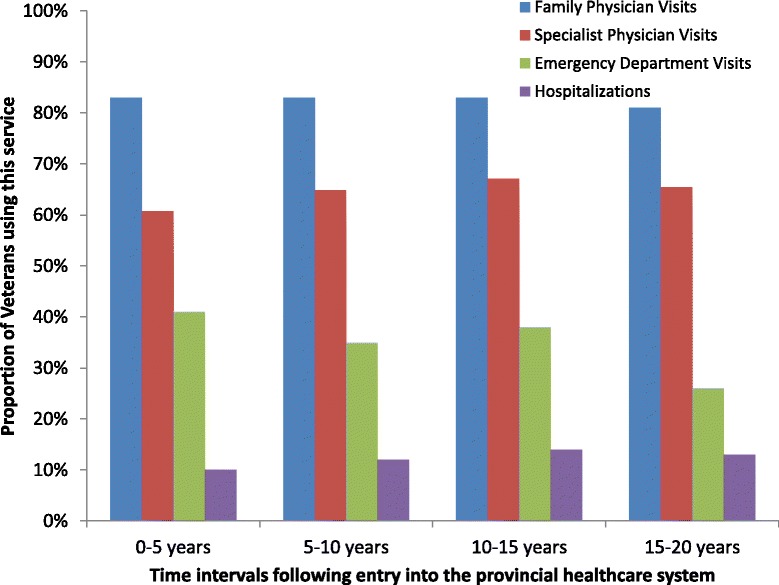
The proportion of Veterans in Ontario accessing a family physician, specialist, the emergency department and requiring hospitalization for non-mental health purposes in the twenty years following entry into the Ontario healthcare system

Following entry into the provincial healthcare system, 82.6 % (CI 82.1-83.1) of Veterans saw their family physician at least once over the first five years following release, 40.8 % (CI 40.2–41.5) went to the emergency department in that same time period and 9.9 % (9.5-10.3) were hospitalized for non-mental health related complaints (Fig. 2). Over the duration of the study, the proportion visiting a family doctor remained stable, while the proportion visiting the emergency department decreased and the proportion hospitalized increased slightly. The average number of physician and inpatient health visits accessed during each time interval is reported in Table 1.

**Table 1:** Overall description of general non-mental health related health services utilization in five year intervals following entry into the Ontario healthcare system

		Time intervals following entry into the provincial healthcare system			
Non-mental health services utilization		0-5 years	5-10 years	10-15 years	15-20 years
		(*N* = 23,818)	(*N* = 15,900)	(*N* = 10,688)	(*N* = 6,191)
Healthcare Encounters					
Family Doctor Visit	Mean (SD)	11.81 (13.24)	13.32 (13.91)	13.60 (14.40)	10.45 (11.29)
	Median (IQR)	8 (4–15)	10 (4–18)	10 (5–18)	7 (3–14)
	Mean (SD)	6.89 (8.78)	7.98 (10.51)	9.13 (10.77)	8.63 (10.12)
	Median (IQR)	4 (2–9)	5 (2–10)	6 (2–12)	5 (2–11)
Emergency Room Visits	Mean (SD)	3.17 (4.58)	2.65 (3.10)	2.69 (3.33)	2.33 (2.31)
	Median (IQR)	2 (1–4)	2 (1–3)	2 (1–3)	2 (1–3)
Hospitalizations	Mean (SD)	1.58 (1.28)	1.61 (1.41)	1.64 (1.54)	1.65 (1.33)
	Median (IQR)	1 (1–2)	1 (1–2)	1 (1–2)	1 (1–2)
Cumulative hospital stay (days)	Mean (SD)	6.93 (13.78)	8.05 (15.58)	8.13 (14.01)	10.63 (18.59)
	Median (IQR)	3 (2–7)	4 (2–8)	4 (2–8)	5 (2–10)

**Fig. 3:**
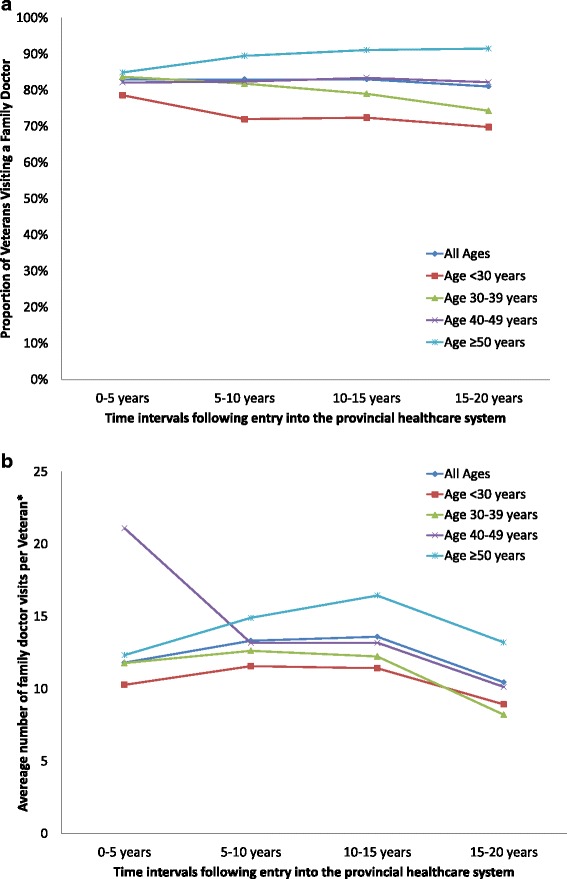
**a** The proportion of Veterans in Ontario visiting a family doctor for non-mental health reasons, stratified by age of the Veteran at entry into the Ontario healthcare system. **b** The average number of non-mental health family doctor visits by Veterans in Ontario who saw a family doctor, stratified by age of the Veteran at entry into the Ontario healthcare system. * The average was calculated for Veterans using this service at least once over the time interval

Stratifying health services utilization by age of the Veteran at entry into the provincial healthcare system revealed differences in service use and intensity (Additional file 1: Table S1 and Additional file 2: Table S2). In the first five years after entry into the healthcare system, a larger proportion of Veterans aged 50 and older visited a family doctor than Veterans under age 30 (Fig. 3a). This pattern persisted over the time intervals studied. Similarly, in the fifteen years following entry into the system, Veterans who were under 30 when they released, who visited a family doctor went on average 8 times every five years, while Veterans who were 50 and older averaged 12 to 16 visits per person over the same time intervals (Fig. 3b). Again, the average number of visits was highest in the group of Veterans who were 50 and older at the time of release.

**Fig. 4:**
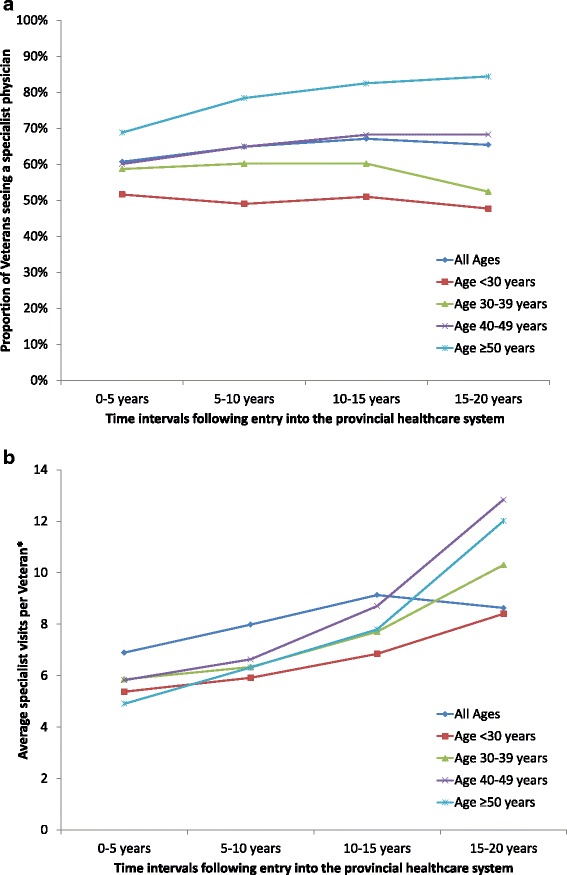
**a** The proportion of Veterans in Ontario visiting a specialist physician for non-mental health reasons, stratified by age of the Veteran at entry into the Ontario healthcare system. **b** The average number of non-mental health specialist physician visits by Veterans in Ontario who saw a specialist physician, stratified by age of the Veteran at entry into the Ontario healthcare syste. * The average was calculated for Veterans using this service at least once over the time interval

A larger proportion of Veterans in the 50 or older age category (68.6 %) saw a non-mental health specialist in the first five years following release compared to the youngest age category (51.6) (Fig. 4a). This pattern persisted and differences in use increased over time as the cohort aged. The average number of visits per Veteran to a non-mental health specialist was lowest in the first five years following release, and increased over time (Fig. 4b).

**Fig. 5:**
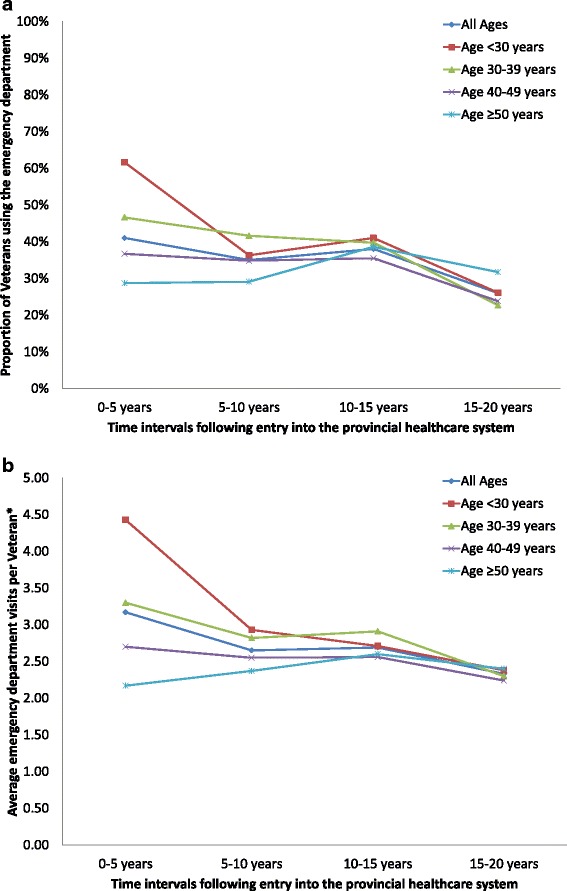
**a** The proportion of Veterans in Ontario accessing the emergency department for non-mental health reasons, stratified by age of the Veteran at entry into the Ontario healthcare system. **b** The average number of emergency department visits for non-mental health reasons by Veterans in Ontario, stratified by age of the Veteran at entry into the provincial healthcare system. * The average was calculated for Veterans using this service at least once over the time interval

Younger Veterans were higher users of emergency departments in the first five years following entry into the provincial healthcare system than older Veterans (Fig. 5a). Almost 61.6 % of Veterans who were under 30 at the time of entry into the system visited the emergency department, compared to 46.6 % of Veterans aged 30–39, 36.7 % of Veterans aged 40–49, and 28.7 % of Veterans 50 and older. The average number of visits per Veteran who used the emergency department was also highest in the younger Veterans (aged 30 or younger) in the first five years following release from service, although this difference attenuated at subsequent time points (Fig. 5b).

Patterns of hospitalization across the time intervals differed according to the age of the Veteran at entry into the provincial healthcare system (Additional file 1: Table S1 and Additional file 2: Table S2). Hospitalization rates decreased over time for Veterans in the two youngest age categories, and increased over time for the two older age categories. Veterans in the oldest age category had the highest rates of inpatient admissions. Among individuals who were hospitalized over the study period, older Veterans had longer cumulative lengths of stay within each time interval than younger Veterans (Additional file 1: Table S1 and Additional file 2: Table S2). The number of hospital days per time interval appeared relatively stable until the 15–20 years from entry into the provincial system. This may be due to the smaller sample sizes within each category for that period of follow-up, as the median values remained relatively unchanged.

## Discussion

This study provides the first general overview of Veteran non-mental health services utilization within the Ontario public healthcare system. Veterans residing in Ontario are steady and consistent users of the provincial healthcare system from the time they enter the system following release from service. Independent of age, a high proportion of Veterans visited a family doctor in the first five years following entry into the provincial healthcare system. Patterns of non-mental health services utilization in Canadian Veterans appear to change over time, depend on the service being provided, and differ according to the age of the Veteran at entry into the provincial healthcare system. While there is high engagement of non-mental health primary care across all age categories, there is a large difference in the use of emergency department visits between younger and older Veterans. This may suggest that either young Veterans have more non-mental health medical crises despite primary care physician involvement, or that they are not getting enough healthcare from regular primary care physician engagement. Overall, non-mental healthcare services are time-dependent and requirements for some healthcare services differ between younger and older Veterans.

Family physicians are considered a key point of contact within the Canadian public healthcare system. The *Veteran File* series published in the Canadian Family Physician in collaboration with Veterans Affairs Canada documents the important role primary healthcare clinicians play in addressing the unique needs of Canadian Veterans living in Ontario [13]. In our study, we saw that 83 % of Veterans in Ontario visited a family physician at least once in the first five years following release (ranging from 79 % in Veterans under 30 years old to 85 % in older Veterans older than 50 years old) and observed these rates are relatively stable over time. The ICES primary care atlas reported only 45 % of individuals aged 20–39 and 39 % in those age 40 and older visited a primary care doctor annually [10], which indicates that Veterans are higher users of primary care services in the first five years following release from the CAF. These rates are consistent with the self-reported findings from the Life After Service Study in 2013 that reported 81 % (79–83 %) of Veterans have a family doctor [4]. This is also consistent with reported American Veteran health services utilization rates [14]. Veterans in Ontario who accessed primary care visited a family doctor 10 or more times over each 5 year period, almost half the rate reported in the US with a reported average rate of 3.5–3.8 visits per Veteran per year [14]. The physician visit and hospitalization count data were right skewed, with higher mean values than medians. This indicates that a few individuals are likely heavier users of these health services than the majority of Veterans, similar to non-Veteran service utilization in Ontario [15, 16].

Within users of primary care, older Veterans had a higher average number of family physician visits than younger Veterans. These differences are expected given the relationship between aging and disease, differences in the need for chronic disease care between the populations, and a potentially longer history of an occupationally demanding career [4, 17]. Understanding how burden of chronic disease, and occupational injuries may explain these higher utilization rates is an important step in interpreting these data and mobilizing the information to inform policy. In addition, understanding how previous usual healthcare utilization patterns may differ for active CAF members, where individuals often interact routinely with the CAF health system will be critical to understanding current use. Follow up on usual healthcare utilization patterns for RCMP Veterans will also be critical.

Utilization of public health services including inpatient services and emergency departments also appear high within each Veteran age category compared to what is known about the general Ontario population. In Ontario, the age-sex standardized rate of acute inpatient admission was approximately 7 per 100 people in 2010–2011 [18]. In our study, on average 10 % of Veterans were hospitalized in any of the five-year time frames following release, with those over age 50 being hospitalized more frequently. In 2000, 20 % of Ontarians visited the emergency department at least once, and over 20 % were from individuals less than 20 years old [19]. In our study, more than 30 % of Veterans visited the emergency department in the first five years following release. These results are especially interesting considering that we have not included mental-health related visits in our utilization results. Identifying an appropriate comparator group from the civilian population will be instrumental in understanding how the patterns of health services utilization of Veterans compare to the general population.

Gender and age are both highly associated with use of healthcare services and only age was considered in our presentation of healthcare utilization. Although female Veterans may have experienced different healthcare issues or gaps in healthcare related to their military service [20–23], fewer studies focus on the health and healthcare utilization of female Veterans due to small sample sizes. Because, on average 15 % of the CAF serving members are female and 20 % of the RCMP are female [24], the large numbers in this cohort will allow us to study male and female Veterans separately in the future. Canadian research studies describing age and sex standardized annual health services utilization rates to contrast with the general population are needed to develop a better understanding of how Canadian Veterans living in Ontario use the public healthcare system.

### Limitations

The exact denominator of Veterans in Ontario is unavailable and we cannot be certain that all Veterans residing in Ontario are included in our cohort. Veterans may not self-identify during their application for OHIP coverage. VAC release statistics provide assurance that the number of releasing CAF members and demographic profile of Veterans who settle in Ontario support the population-based nature of our cohort and support that these results are likely generalizable to other Canadian Veterans [25]. We have further discussed the representativeness of the cohort elsewhere [9].

These administrative healthcare data provide the first picture of health services use for Veterans, but do not cover all possible health care services provided. For example, this study does not cover healthcare services provided to eligible Veterans via supplementary healthcare coverage and other benefits from Veterans Affairs Canada. In addition, this study does not cover privately insured services, those services not provided through a hospital (e.g., physiotherapy, occupational therapy or chiropractic services) or by nurse practitioners. However, this is the first description of public health services use and these established data are consistently used to describe healthcare utilization in the Ontario population.

We have not reported direct comparisons with the general Ontario population, given concerns about the healthy worker effect. Occupational cohorts are typically healthier than the general population, given that they are able to maintain employment. However, Veterans who leave the CAF or RCMP may be the result of medical releases and may actually have a greater need for services than the general population. Unfortunately data on the type of medical release are not currently available. Type of release will likely have a greatest impact on the utilization rates of Veterans who served for the shortest and longest periods of time. Work is ongoing to identify a more tailored control group for direct comparisons necessary, using recently published work from the Canadian Armed Forces as guidance [26].

## Conclusions

This study provides the first description of the of the public health services utilization of a cohort of Canadian Veterans living in Ontario. The results fill a gap in our knowledge about how CAF and RCMP Veterans transition to the public healthcare system and provide a picture of how healthcare services are used following release. This study finds consistent use of non-mental health services by Veterans, particularly in the first five years following release from the CAF & RCMP and regardless of age. The implications of this work will inform the planning and delivery of support to Veterans in Ontario. Future research could include additional information on publicly funded home care, long term care and rehabilitation institutions and provide gender and age specific rates of healthcare utilization more comparable to those reported for the general Canadian population. Future health services research for Veterans should also target understanding the provincial costs of caring for Veterans in addition to the costs of federally mandated programs in an aging population. The development of predictive models to identify high cost or high health services users within the Veteran population could also be useful for future healthcare planning in the provincial and federal government.

## Abbreviations

CAF, Canadian Armed Forces; CIHI-DAD, Canadian Institute for Health Information Discharge Abstract Database; ED, emergency department; ICD, international classification of disease; ICES, Institute for Clinical Evaluative Sciences; IQR, interquartile range; LHIN, Local Health Integration Network; MOHLTC, Ministry of Health and Long-Term Care; NACRS, national ambulatory care reporting system; OHIP, Ontario Health Insurance Plan; RCMP, Royal Canadian Mounted Police; RPDB, registered persons database; SD, standard deviation; VAC, Veterans Affairs Canada

## Additional files

Additional file 1: Table S1. The proportion of Veterans in Ontario accessing a family physician, the emergency department and requiring hospitalization in the twenty years following entry into the provincial healthcare system, stratified by age at entry into the provincial healthcare system. (DOC 62 kb) Additional file 2: Table S2. The average number of health services used by Veterans in Ontario in the twenty years following entry into the provincial healthcare system, stratified by age at entry into the provincial healthcare system stratified by age at entry into the provincial healthcare system. (DOC 51 kb)

## References

[1] LeBlanc D (2012). Budget bill to upend RCMP health care.

[2] Toews THV. Royal Canadian Mounted Police Report on Plans and Priorities 2013–2014. vol. 2015: Ministry of Public Safety; 2013. http://www.rcmp-grc.gc.ca/en/royal-canadian-mounted-police-2013-14-departmental-performance-report

[3] Health Canada. Canada Health Act- Annual Report 2012–2013. In. Ottawa: Health Canada; 2013. http://www.hc-sc.gc.ca/hcs-sss/alt_formats/pdf/pubs/cha-ics/2013-cha-lcs-ar-ra-eng.pdf

[4] Thompson JM, Van Til L, Poirier A, Sweet J, McKinnon K, Sudom K, Dursun S, Pedlar D, Canada RDVA (2014). Health and well-being of Canadian armed forces veterans: findings from the 2013 life after service survey.

[5] Veterans Affairs Canada: Five Year Strategic Plan 2009–2014 [http://www.veterans.gc.ca/eng/about-us/reports/five-year-plans/2009-2014#s12]

[6] Thompson JM, MacLean MB, Van Til L, Sudom K, Sweet J, Poirier A, Adams J, Horton V, Campbell C, Pedlar D, Analysis VACDGMPRa (2011). Survey on Transition to Civilian Life: Report on Regular Force Veterans.

[7] Tien HCN, Acharya S, Redelmeier DA (2010). Preventing deaths in the Canadian military. Am J Prev Med.

[8] Mahar AL, Aiken A, Groome P, Kurdyak P (2015). A new resource to study the health of Veterans in Ontario. J Military Veteran Family Health.

[9] Mahar AL, Aiken AB, Kurdyak P, Whitehead M, Groome PA (2016). Description of a longitudinal cohort to study the health of Canadan Veterans living in Ontario. J Military Veteran Family Health.

[10] Jaakkimainen L, Schultz SE, Klein-Geltink J, Thiruchelvam D, Kopp A, Jaakkimainen L, Upshur R, Klein-Geltink JE, Leong A, Maaten S, Schultz SE, Wang L (2006). Ambulatory Physician Care for Adults. Primary Care in Ontario: ICES Atlas.

[11] Steele LS, Glazier RH, Lin E, Evans M (2004). Using administrative data to measure ambulatory mental health service provision in primary care. Med Care.

[12] Porta MS, International Epidemiological A (2008). A dictionary of epidemiology.

[13] Thompson JM, Chiasson R, Pedlar D (2008). Launch of the veteran health files series. Can Family Phys.

[14] Liu CF, Bryson CL, Burgess JF, Sharp N, Perkins M, Maciejewski ML (2012). Use of outpatient care in VA and medicare among disability-eligible and age-eligible veteran patients. BMC Health Serv Res.

[15] Rais S, Nazerian A, Ardal S, Chechulin Y, Bains N, Malikov K (2013). High-cost Users of Ontario's Healthcare Services. Healthc Policy.

[16] Rosella LC, Fitzpatrick T, Wodchis WP, Calzavara A, Manson H, Goel V (2014). High-cost Health Care Users in Ontario, Canada: Demographic, Socioeconomic, and Health Status Characteristics. BMC Health Serv Res..

[17] Stroupe KT, Smith BM, Hogan TP, St Andre JR, Pape T, Steiner ML, Proescher E, Huo Z, Evans CT (2013). Healthcare utilization and costs of Veterans screened and assessed for traumatic brain injury. J Rehabil Res Dev.

[18] Trends in acute inpatient hospitalizations and day surgery visits in Canada, 1995–1996 to 2005–2006. In: Analysis in Brief. Canadian Institute for Health Information; 2007: 1–26

[19] Chan BTB, Schull MJ, Schultz SE (2001). Emergency department services in Ontario.

[20] Di Leone BA, Wang JM, Kressin N, Vogt D: Women’s Veteran Identity and Utilization of VA Health Services. Psychol Serv. 2016;13(1):60–8 10.1037/ser0000021 25729892

[21] Hoff RA, Rosenheck RA (1998). Female veterans’ use of department of veterans affairs health care services. Med Care.

[22] Koo KH, Madden E, Maguen S (2015). Race-ethnicity and gender differences in VA health care service utilization among U.S. Veterans of recent conflicts. Psychiatr Serv.

[23] Washington DL, Bean-Mayberry B, Riopelle D, Yano EM (2011). Access to care for women veterans: delayed healthcare and unmet need. J Gen Intern Med.

[24] National Program Evaluation Services RCMP: Gender-based assessment. In.; 2012.

[25] MacLean MB, Poirier A, O’Connor T, Canada VA (2011). Province of residence at release and post-release- Data from the income study.

[26] Rusu C, Zamorski MA, Boulos D, Garber BG (2016). Prevalence comparisons of past-year metnal disorders and suicidal behaviours in the Canadian Armed Forces and the Canadian general population. Canadian Journal of Psychiatry.

