# Supplementary material for: A descriptive analysis of medical health services utilization of Veterans living in Ontario: a retrospective cohort study using administrative healthcare data
Source: BMC Health Serv Res. 2016 Aug 4;16:351. doi: 10.1186/s12913-016-1596-y (PMC4973105; doi:10.1186/s12913-016-1596-y)
Supplement: Additional file 1: Table S1. — The proportion of Veterans in Ontario accessing a family physician, the emergency department and requiring hospitalization in the twenty years following entry into the provincial healthcare system, stratified by age at entry into the provincial healthcare system. (DOC 62 kb) [file 12913_2016_1596_MOESM1_ESM.doc]

**Additional file 1: Table S1 The proportion of Veterans in Ontario accessing a family physician, the emergency department and requiring hospitalization in the twenty years following entry into the provincial healthcare system, stratified by age at entry into the provincial healthcare system.**

| **Non-Mental Health Services Utilization** | **Age Category** | **0-5 Years** | | | | **5-10 Years** | | | | **10-15 Years** | | | | **15-20 Years** | | | |
| --- | --- | --- | --- | --- | --- | --- | --- | --- | --- | --- | --- | --- | --- | --- | --- | --- | --- |
|  |  | **N** | **%** | **LCL** | **UCL** | **N** | **%** | **LCL** | **UCL** | **N** | **%** | **LCL** | **UCL** | **N** | **%** | **LCL** | **UCL** |
| **Family Physician Visit** | <30 years | 2947 | 78.6 | 77.3 | 79.9 | 1579 | 72.0 | 70.1 | 73.8 | 1046 | 72.4 | 70.1 | 74.7 | 623 | 69.8 | 66.8 | 72.8 |
|  | 30-39 years | 4530 | 83.7 | 82.7 | 84.6 | 3169 | 81.8 | 80.6 | 83.0 | 2361 | 79.0 | 77.5 | 80.4 | 1238 | 74.3 | 72.2 | 76.4 |
|  | 40-49 years | 7033 | 82.1 | 81.3 | 82.9 | 4817 | 82.4 | 81.4 | 83.3 | 3042 | 83.4 | 82.2 | 84.6 | 1612 | 82.2 | 80.5 | 83.9 |
|  | ≥ 50 years | 5164 | 84.9 | 84.0 | 85.8 | 3564 | 89.5 | 88.6 | 90.5 | 2373 | 91.1 | 90.0 | 92.2 | 1528 | 91.5 | 90.2 | 92.8 |
|  |  |  |  |  |  |  |  |  |  |  |  |  |  |  |  |  |  |
| **Specialist Physician Visit** | <30 years | 1934 | 51.6 | 51.0 | 52.2 | 1074 | 49.0 | 46.9 | 51.0 | 736 | 51.0 | 48.4 | 53.5 | 426 | 47.7 | 44.4 | 51.0 |
|  | 30-39 years | 3181 | 58.7 | 58.1 | 59.4 | 2331 | 60.2 | 58.6 | 61.7 | 1800 | 60.2 | 58.4 | 62.0 | 873 | 52.4 | 50.0 | 54.8 |
|  | 40-49 years | 5148 | 60.1 | 59.5 | 60.7 | 3794 | 64.9 | 63.6 | 66.1 | 2489 | 68.2 | 66.7 | 69.7 | 1340 | 68.3 | 66.2 | 70.4 |
|  | ≥ 50 years | 4187 | 68.8 | 68.2 | 69.4 | 3123 | 78.4 | 77.2 | 79.7 | 2148 | 82.5 | 81.0 | 83.9 | 1410 | 84.4 | 82.7 | 86.2 |
|  |  |  |  |  |  |  |  |  |  |  |  |  |  |  |  |  |  |
| **Emergency Room Visits** | <30 years | 2310 | 61.6 | 60.1 | 63.2 | 797 | 36.3 | 34.3 | 38.3 | 592 | 41.0 | 38.5 | 43.5 | 233 | 26.1 | 23.2 | 29.0 |
|  | 30-39 years | 2526 | 46.6 | 45.3 | 48.0 | 1613 | 41.6 | 40.1 | 43.2 | 1187 | 39.7 | 37.9 | 41.5 | 378 | 22.7 | 20.7 | 24.7 |
|  | 40-49 years | 3140 | 36.7 | 35.6 | 37.7 | 2037 | 34.8 | 33.6 | 36.0 | 1296 | 35.5 | 34.0 | 37.1 | 467 | 23.8 | 21.9 | 25.7 |
|  | ≥ 50 years | 1748 | 28.7 | 27.6 | 29.9 | 1159 | 29.1 | 27.7 | 30.5 | 1005 | 38.6 | 36.7 | 40.4 | 530 | 31.7 | 29.5 | 34.0 |
|  |  |  |  |  |  |  |  |  |  |  |  |  |  |  |  |  |  |
| **Hospitalizations** | <30 years | 389 | 10.4 | 9.4 | 11.4 | 217 | 9.9 | 8.6 | 11.1 | 107 | 7.4 | 6.1 | 8.8 | 48 | 5.4 | 3.9 | 6.9 |
|  | 30-39 years | 530 | 9.8 | 9.0 | 10.6 | 375 | 9.7 | 8.7 | 10.6 | 284 | 9.5 | 8.4 | 10.5 | 135 | 8.1 | 6.8 | 9.4 |
|  | 40-49 years | 764 | 8.9 | 8.3 | 9.5 | 651 | 11.1 | 10.3 | 11.9 | 486 | 13.3 | 12.2 | 14.4 | 245 | 12.5 | 11.0 | 14.0 |
|  | ≥ 50 years | 677 | 11.1 | 10.3 | 11.9 | 651 | 16.3 | 15.2 | 17.5 | 615 | 23.6 | 22.0 | 25.2 | 354 | 21.2 | 19.2 | 23.2 |
