# Supplementary material for: A descriptive analysis of medical health services utilization of Veterans living in Ontario: a retrospective cohort study using administrative healthcare data
Source: BMC Health Serv Res. 2016 Aug 4;16:351. doi: 10.1186/s12913-016-1596-y (PMC4973105; doi:10.1186/s12913-016-1596-y)
Supplement: Additional file 2: Table S2. — The average number of health services used by Veterans in Ontario in the twenty years following entry into the provincial healthcare system, stratified by age at entry into the provincial healthcare system stratified by age at entry into the provincial healthcare system. (DOC 51 kb) [file 12913_2016_1596_MOESM2_ESM.doc]

Additional file 2: Table S2: The average number of health services used by Veterans in Ontario in the twenty years following entry into the provincial healthcare system, stratified by age at entry into the provincial healthcare system.

| **Non-Mental Health Services Utilization** | **Age Category** | **0-5 Years**  (N=23,818) | | **5-10 Years**  (N=15,900) | | **10-15 Years**  (N=10,688) | | **15-20 Years**  (N=6,191) | |
| --- | --- | --- | --- | --- | --- | --- | --- | --- | --- |
|  |  | **Mean (SD)** | **Median (IQR)** | **Mean (SD)** | **Median (IQR)** | **Mean (SD)** | **Median (IQR)** | **Mean (SD)** | **Median (IQR)** |
| **Healthcare Encounters** | | | | | | | | | |
| **Family Physician Visits** | <30 years | 8.08 (14.01) | 4 (1-10) | 8.33 (14.68) | 4 (0-11) | 8.27 (13.58) | 4 (0-11) | 6.23 (10.13) | 3 (0-9) |
|  | 30-39 years | 11.78 (14.15) | 8 (4-15) | 12.63 (13.68) | 9 (4-16) | 12.24 (15.11) | 8 (4-15) | 8.21 (10.38) | 5 (2-10) |
|  | 40-49 years | 12.10 (12.88) | 8 (4-16) | 13.17 (13.42) | 10 (4-18) | 13.19 (13.33) | 10 (5-18) | 10.13 (10.88) | 7 (3-13) |
|  | ≥ 50 years | 12.33 (11.63) | 9 (5-17) | 14.91 (13.52) | 12 (6-20) | 16.45 (14.42) | 14 (7-22) | 13.21 (11.92) | 10 (5-17) |
| **Specialist Physician Visits** | <30 years | 5.37 (7.47) | 3 (1-6) | 5.85 (8.45) | 3 (1-7) | 5.82 (8.25) | 3 (2-7) | 4.90 (6.09) | 3 (1-6) |
|  | 30-39 years | 5.91 (8.36) | 3 (2-7) | 6.33 (7.98) | 4 (2-8) | 6.63 (9.06) | 4 (2-8) | 6.31 (8.60) | 4 (2-7) |
|  | 40-49 years | 6.84 (8.81) | 4 (2-8) | 7.70 (11.52) | 4 (2-9) | 8.70 (10.53) | 5 (2-11) | 7.79 (8.74) | 5 (2-10) |
|  | ≥ 50 years | 8.40 (9.37) | 5 (2-11) | 10.30 (11.10) | 7 (3-14) | 12.84 (12.00) | 9 (4-17) | 12.01 (12.05) | 8 (4-15) |
| **Emergency Room Visits** | <30 years | 4.43 (5.33) | 3 (1-6) | 2.93 (3.98) | 2 (1-3) | 2.71 (3.04) | 2 (1-3) | 2.38 (2.23) | 1 (1-3) |
|  | 30-39 years | 3.30 (4.12) | 2 (1-4) | 2.82 (3.40) | 2 (1-3) | 2.91 (4.18) | 2 (1-3) | 2.30 (2.29) | 1 (1-3) |
|  | 40-49 years | 2.70 (5.09) | 2 (1-3) | 2.55 (2.81) | 2 (1-3) | 2.56 (3.09) | 2 (1-3) | 2.24 (2.32) | 1 (1-2) |
|  | ≥ 50 years | 2.17 (2.09) | 1 (1-2) | 2.37 (2.35) | 2 (1-3) | 2.60 (2.56) | 2 (1-3) | 2.40 (2.37) | 2 (1-3) |
| **Hospitalizations** | <30 years | 1.56 (1.00) | 1 (1-2) | 1.41 (0.72) | 1 (1-2) | 1.38 (0.90) | 1 (1-2) | 1.56 (2.36) | 1 (1-1) |
|  | 30-39 years | 1.52 (1.30) | 1 (1-2) | 1.55 (1.74) | 1 (1-1) | 1.58 (1.27) | 1 (1-2) | 1.60 (1.32) | 1 (1-2) |
|  | 40-49 years | 1.56 (1.32) | 1 (1-2) | 1.63 (1.47) | 1 (1-2) | 1.64 (2.06) | 1 (1-2) | 1.67 (1.33) | 1 (1-2) |
|  | ≥ 50 years | 1.66 (1.35) | 1 (1-2) | 1.67 (1.29) | 1 (1-2) | 1.71 (1.23) | 1 (1-2) | 1.67 (1.14) | 1 (1-2) |
| **Cumulative hospital stay (days)** | <30 years | 4.89 (7.48) | 3 (2-5) | 4.72 (8.62) | 3 (2-5) | 4.56 (6.25) | 3 (1-5) | 7.77 (23.62) | 3 (2-5) |
|  | 30-39 years | 5.95 (11.93) | 3 (2-5) | 6.70 (17.01) | 3 (1-5) | 6.83 (12.89) | 3 (1-7) | 9.93 (17.68) | 4 (2-9) |
|  | 40-49 years | 6.86 (11.80) | 3 (2-7) | 8.31 (15.96) | 4 (2-8) | 8.17 (15.63) | 4 (2-8) | 10.92 (17.97) | 5 (3-10) |
|  | ≥ 50 years | 8.95 (18.83) | 4 (2-9) | 9.70 (15.94) | 5 (3-11) | 9.31 (13.99) | 5 (3-11) | 11.08 (18.63) | 5 (3-11) |
